# Nutrition education and cooking workshops for families of children with cancer: a feasibility study

**DOI:** 10.1186/s40795-019-0319-2

**Published:** 2019-11-19

**Authors:** S. Beaulieu-Gagnon, V. Bélanger, C. Meloche, D. Curnier, S. Sultan, C. Laverdière, D. Sinnett, V. Marcil

**Affiliations:** 1 Department of Nutrition, Université de Montréal, Montreal, QC, Canada; 2 Research Center of Sainte-Justine University Health Center, 3175 Côte Sainte-Catherine room 4.17.006, Montreal, QC H3T 1C5 Canada; 3 Department of Kinesiology, Université de Montréal, Montreal, QC Canada; 4 Department of Psychology, Université de Montréal, Montreal, QC Canada; 5 Division of Hematology-Oncology, Sainte-Justine University Health Center, Montreal, QC Canada; 6 Department of Pediatrics, Université de Montréal, Montreal, QC Canada

**Keywords:** Nutrition workshops, Culinary demonstration, Process evaluation, Childhood cancer, Parents

## Abstract

**Background:**

Changes in food intake are common in children with cancer and are often caused by nausea and perturbations in sense of taste. The VIE (Valorization, Implication, Education) study proposes family-based nutrition and cooking education workshops during childhood cancer treatments. Process evaluation during implementation allows to assess if the intervention was delivered as planned and to determine its barriers and facilitators. The study objective was to describe the implementation process of a nutrition education and cooking workshop program for families of children actively treated for cancer in a non-randomized non-controlled feasibility study.

**Methods:**

Six open-to-all in-hospital workshops were offered on a weekly basis during a one-year implementation phase. We collected qualitative and quantitative data using field notes and activity reports completed by the registered dietician facilitator; surveys and questionnaires fulfilled by the workshop participants and by the families enrolled in the VIE study. Field notes were used to collect only qualitative data. Survey respondents (*n* = 26) were mostly mothers (*n* = 19, 73%). Children’s mean age was 7.80 (± 4.99) years and the mean time since diagnosis was 7.98 (± 0.81) months. Qualitative data were codified using hybrid content analysis. The first deductive analysis was based on the Steckler & Linnan concepts. Subthemes were then identified inductively. Quantitative data were presented with descriptive statistics.

**Results:**

Workshop attendance was low (17 participants over 1 year) and 71% of the planned workshops were cancelled due to lack of participants. The principal barriers to participation referred the child’s medical condition, parental presence required at the child’s bedside and challenges related to logistics and time management. The level of interest in the topics addressed was found high or very high for 92% of the participants. The themes that were perceived as the most useful by parents were related to the child’s specific medical condition.

**Conclusions:**

Despite high interest, workshops delivered in a face-to-face format were poorly feasible in our sample population. This supports the need to develop educational programs in pediatric oncology using strategies and delivery formats that address the major barriers for participation encountered by families.

## Background

During treatment of childhood and adolescent cancer, many factors may influence food intake. Among others, changes in sense of taste and gastrointestinal side effects can alter children’s appetite and modify their preferences [1–4]. It has been reported that children undergoing cancer treatment often experience changes in their food preferences towards fat and savory foods [2, 4, 5]. Parents have stated that their child’s cravings and pickiness are very difficult challenges to manage and that they rarely know which strategies are best to use during these phases [1].

Children with cancer are both at risk of malnutrition and excessive weight gain during treatments. Studies showed that the use of corticosteroids can lead to higher energy intake compared to off-steroid periods and to healthy controls [6, 7]. On the other hand, a decreased appetite can impair nutritional status that is associated with lower tolerance to treatments and to a higher prevalence of infections [8].

The eating habits acquired during treatments are maintained throughout survivorship [9–11]. They were found not different to those of the general population and thus non-favorable to prevent cardiovascular and other lifestyle-influenced diseases [9]. Considering the growing literature showing that childhood cancer survivors have a higher risk to develop health complications such as dyslipidemia and the metabolic syndrome [12, 13], nutritional interventions and education are promising avenues to reduce the risk of long-term medical sequelae.

Most of the existing nutrition education interventions in childhood cancer were designed for patients at the end of their treatment or for survivors [14–20] and few have included a culinary component [21, 22]. However, intervening at the end of cancer treatment might be too late to reverse the acquired food habits [18]. While this supports the need for early interventions, their feasibility in the context of a recent diagnosis and initiation of treatment is unknown.

Process evaluation allows to better understand and explain the success or failure of a program. Barriers and facilitators can be raised, therefore making it possible to adequately attribute outcomes to the intervention rather than to its implementation [23]. The components proposed by *Steckler and Linnan* for public health interventions are *fidelity*, *dose delivered*, *dose received*, *context*, *recruitment* and *reach* [23].

Combining multiple methods of data collection (quantitative and qualitative) can enhance the richness of the further interpretation [23–27]. In this study, we describe the implementation process of an in-hospital nutritional and culinary education workshop program for families of children with cancer. Our main aim was to assess the feasibility of in-hospital face-to-face workshops early after the child’s diagnosis. Our secondary objective was to identify the facilitators and challenges in workshop participation encountered by families.

## Methods

### Description of the nutritional and culinary workshops

This work is part of the VIE (Valorization, Implication, Education) multidisciplinary study including nutritional, psychological and physical activity interventions at Sainte-Justine University Health Center (SJUHC) in Montreal, Quebec, Canada. The development of the curriculum of the VIE nutritional and culinary workshops has been described elsewhere [22]. Briefly, the workshops were developed and tested following an 8-step iterative process, including a review of the literature and consultations with a steering committee. The workshops consisted in weekly culinary demonstrations coupled with nutritional key messages and were destined to parents of children with cancer and their relatives. Patients and siblings were also invited to participate. Activities such as drawing and puzzles were planned for young children. Workshops were delivered by a registered dietician, the principal facilitator, and by a chef. They addressed themes related to nutrition and childhood cancer and to general principles of healthy eating. Workshops were independent from each other and included information related to the prevention of foodborne infections for immunocompromised patients. The workshops took place two floors below the inpatient ward of the SJUHC Hematology-Oncology Division. Families could attend the workshops either when the child was hospitalized or was an outpatient. The themes of the 6 workshops were: 1) “Meal fortification”; 2) “Changes in taste during cancer therapy and their impact on children”; 3) “Adapting diet to eating-related side effects of treatments”; 4) “Nutritional support”; 5) “Mediterranean diet and health” and; 6) “Planning quick and economic meals”. The VIE study was approved by the SJUHC Institutional Review Board.

#### Participants

**Fig. 1:**
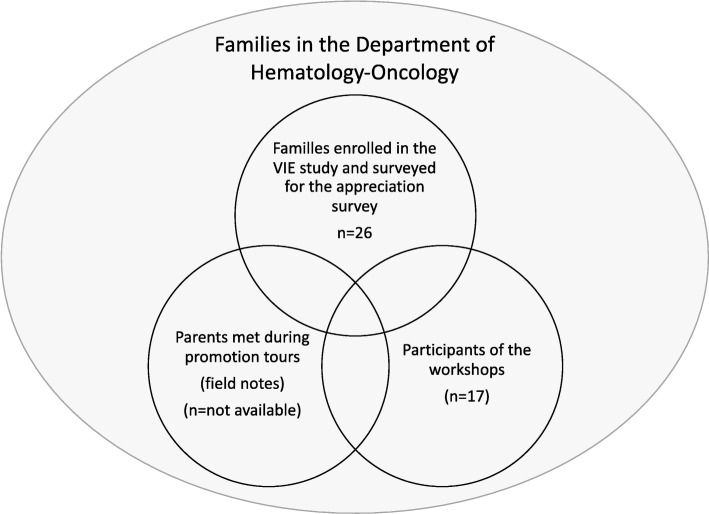
Description of the different samples in the workshop feasibility study

Participants of the workshops included families recruited as part of the VIE study, as well as other families visiting the Division of Hematology-Oncology. In this study, the term “family” describes the family unit surrounding the child, and refers mainly to the child and/or his parents. Workshop participation was voluntary. Families enrolled in the VIE study were surveyed to sought their appreciation, perceived relevance and utility of the themes, and of the workshop schedule. Field notes were collected from a convenience sample comprised of families present in the common areas of the outpatient and inpatient clinics. This included families enrolled, or not, in the VIE study as well as workshop participants and non-participants. Figure 1 illustrates the study participants and the tools used for data collection. Table 1 describes the cancer diagnoses at admission in the Division of Hematology-Oncology of SJUHC over one year.

**Table 1:** Diagnoses of newly admitted patients in the Division of Hematology-Oncology

Hematological cancer	57
Acute lymphoblastic leukemia	30
Acute myeloid leukemia	6
Chronic myeloid leukemia	1
Lymphoma	20
Brain tumor	16
Astrocytoma	3
Brain stem glioma	3
Brain tumor	2
Embryonic tumor with multilayer rosettes	1
Ependynoma	1
Gliobastoma	1
Glioma	1
Médulloblastoma	3
Oligodendroglioma	1
Solid tumor	43
Carcinoma	2
Dermatofibrosarcoma	1
Ewing sarcoma	4
Germinoma	3
Hepatoblastoma	1
Hepatocarcinome	1
Histiocytosis	2
Neuroblastoma	11
Osteosarcoma/bone tumors	6
Retinoblastoma	4
Rhabdomyosarcoma	2
Sarcoma	1
Teratoma	1
Wilms tumor/renal tumors	4
Benign tumor	2
Others	22
Total	140

### Study design and measures

Based on the *Steckler and Linnan* framework [23] and on the structure proposed by Saunders et al. [27], the process evaluation was based on the exhaustive description of the program. The data collection methods included activity reports, field notes, participant questionnaires, an implementation checklist and a survey. The questions adressed in the tools used to evaluate the implementation process are detailed in Table 2. Discussions with participants were not recorded. The implementation of the workshops occurred on a 12-month period (March 2018 to March 2019). The workshops were scheduled once a week, every week, during a one-year period, except during the Christmas Holidays (2 weeks) and during the facilitator’s personal vacations (3 weeks) or absences for attending conferences (2 weeks); therefore a total of 45 workshops were scheduled.

**Table 2:** Data collected with the different tools for evaluation of the implementation process

Tools	Data collected	Responsible for completing the tool
Participant questionnaire	1. Participant’s relationship with patient	Workshop participants
	2. Perception of knowledge acquisition	
	3. Perceived utility of the recipes and advices	
	4. Recommendations	
	5. Additional comments	
Activity report	1. Identification of the facilitators	Registered dietician facilitator
	2. Identification of the theme presented	
	3. Time and duration of the workshop	
	4. Number of participants	
	5. Divulgation of the nutritional messages as planned	
	6. Challenges and facilitators	
	7. Facilitator’s perception of participants’ interest	
	8. Questions from participants	
	9. Proposed modifications to workshop delivery and content	
	10. Obstacles related to language	
	11. Number of participant questionnaires completed and flyers distributed	
	12. Time required by participant(s) to complete the questionnaire and questions related its completion	
Field notes	1. Notes from the facilitator during activity promotion	Registered dietician facilitator
Appreciation survey	1. Participant’s relationship with patient	Families enrolled in the VIE study
	2. Awareness of the workshops and how they learned about it	
	3. Best time for attending to workshop	
	4. Food tasting as an incentive for participation	
	5. Reason for not attending a workshop	
	6. Preferred approach to disseminate nutritional information	
	7. Perceived utility of the workshop content	
	8. Other comments	

#### Activity report

After each workshop, the facilitator filled an activity report to assess the difficulties and factors that affected the delivery of the workshop.

#### Field notes

The facilitator inquired a convenience sample of parents on their opinion about the workshops during promotional tours in the Division of Hematology-Oncology. No formal interview guide was used. After discussing with the participants, the dietician facilitator used fieldnotes to summarize their feedback. The facilitator did not record the number of families approached or patients’ diagnosis.

#### Participant questionnaires

After each workshop, participants were encouraged to answer a short questionnaire about their satisfaction, perception of utility and knowledge acquisition. The questionnaires were developed for each workshop by the research team and revised by an expert in the field of program evaluation. The participant questionnaires were only used to assess study feasibility and thus were not validated. The development of the questionnaire is described elsewhere [22]. Briefly, in each workshop, 2 to 3 key messages were delivered and the questions related to perception of knowledge acquisition were in line with each key message. For example, for the key message “*Proteins are essential for tissue growth and repair and to support immune system function*”, the corresponding question was “*I have learned that proteins have an important role in tissue growth during cancer treatments*”. The response options were “*I agree; I disagree; I agree more or less; I already knew this information*”.

#### Observation checklist

An observation checklist on workshop content and participants’ involvement was developed.

#### Appreciation survey

An 8-question survey addressing barriers to participation and interest in the workshop content was developed based on field notes. The appreciation survey was administered by the facilitator to parents enrolled in the VIE study nine months after the beginning of the implementation.

### Analysis

**Fig. 2:**
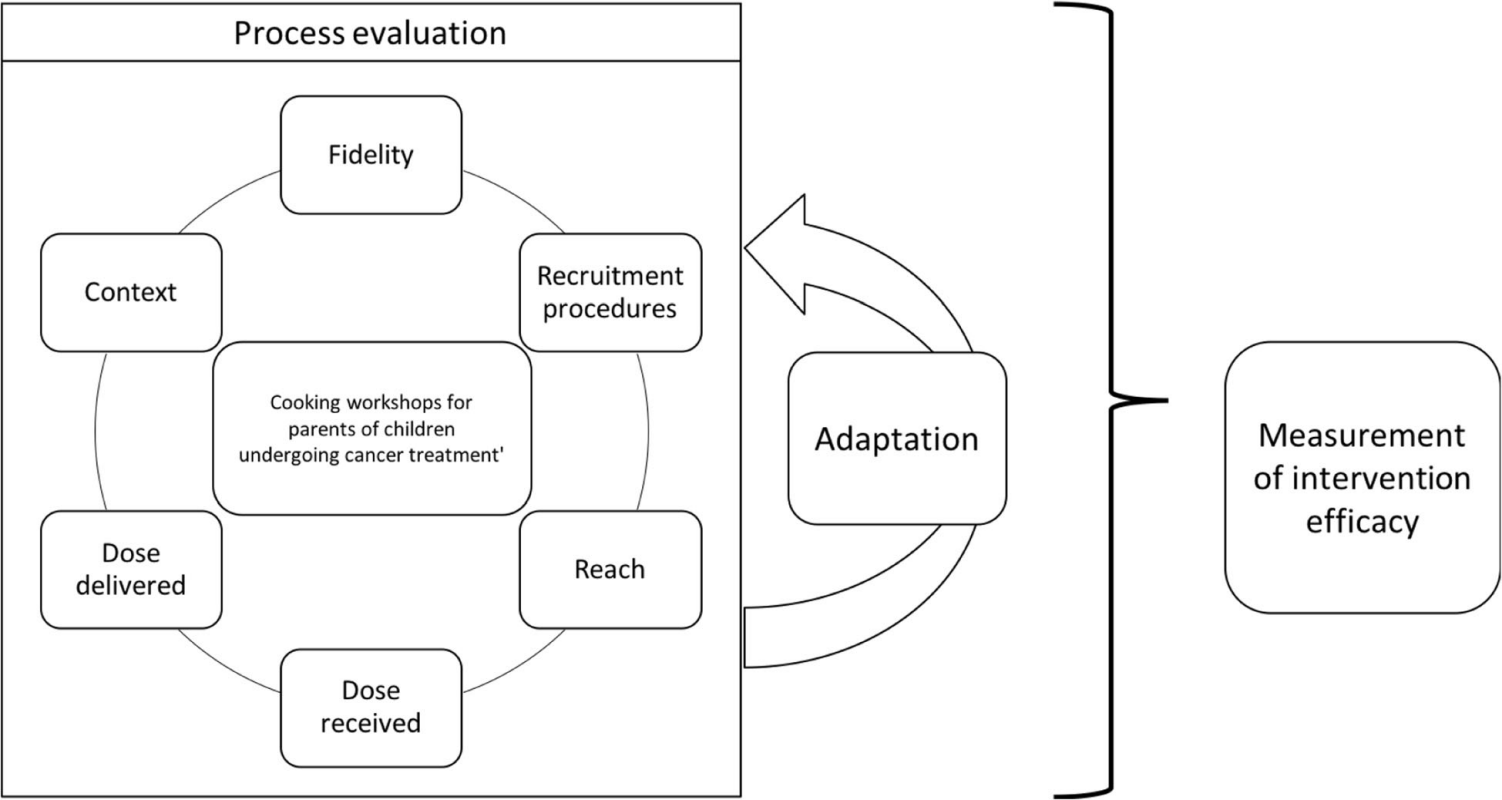
Schematic representation of the workshop implementation process

Data from field notes, activity reports (open-ended questions), participant questionnaires and survey were analyzed with qualitative hybrid analysis combining deductive and inductive analysis [28, 29]. After deconstructing the primary data into 1–2 sentences, all sentences were revised for clarity and to verify that the context of the answer or note was taken into account. The sentences were numbered and were classified per collection methods in an Excel spreadsheet. For the deductive analysis, 453 segments were codified based on the components of the *Steckler and Linnan* framework [23]. The *fidelity* refers to the degree to which an ***intervention*** or program is delivered as intended. The *dose delivered* reflects implementation completeness or the amount of the intervention that is disseminated. The *dose received* refers to the extent to which participants are exposed to the intervention and their satisfaction towards it. The *context* (environmental factors) and *recruitment* procedures are documented for their potential impact on implementation. Assessment of *reach* provides evidence on whether and how the intended audience participates in the intervention or specific intervention components. Figure 2 provides an overview of the framework used for the workshop implementation process.

After, sub-themes were determined for each component by inductive content analysis. All the qualitative segments were codified by S.B.G. For inter-reliability evaluation, the coding was revised by V.M. who was not involved in data collection. Themes were discussed until a consensus was made on the sub-themes. Quantitative data were expressed as descriptive statistics.

## Results

### Workshop delivery and participant characteristics

Workshops were presented in French to a total of 17 participants. Characteristics of the participants are presented in Table 3. Fifteen participants (88%) were parents, of which 11 (65%) were mothers. Four children attended the workshops (3 patients and 1 sibling) of which 2 were too young to answer the questionnaire. Two participants attended more than one workshop.

**Table 3:** Characteristics of the workshop participants

	All participants n = 17
Participants per workshop, mean (SD, range)	1.53 (0.52, 1–2)
Relationship with patient, n (%)	
Mother	11 (65%)
Father	4 (23%)
Patient	2 (12%)
Participants enrolled in the VIE study, n (%)	10 (59%)
Participants who participated to more than one workshop, n (%)	2 (12%)

SD: Standard deviation.

On a total of 45, 13 workshops (29%) were delivered and 32 (71%) were cancelled (Table 4). Over the 12-month implementation period (March 2018 to March 2019), 7 of the workshops delivered (69%) were held with only the facilitator, without the chef (Table 4).

**Table 4:** Characteristics of the workshops as described by the facilitator in the activity reports

Characteristics	Workshops
Workshops cancelled, n (%)	32 of 45 (71%)
Workshops delivered, n (%)	13 (29%)
Meal fortification, n (%)	3 (23%)
Changes in taste during cancer therapy and their impact on children, n (%)	3 (23%)
Adapting diet to eating-related side effects of treatments, n (%)	2 (15%)
Mediterranean diet and health, n (%)	1 (8%)
Planning quick and economic meals, n (%)	4 (31%)
Nutritional support, n (%)	0 (0%)
Workshops delivered without the chef, n (%)	9 (69%)
Duration in minutes, mean (SD, range)	
All workshops	51.4 (12.8, 40–90)
Workshops without the chef	45.9 (4.0, 40–50)
Workshops with children participants, n (%)	4 (31%)
Workshops with 100% of the messages covered, n (%)	7 (54%)
High to very high level of interest as perceived by the facilitator	12 (92%)

SD: Standard deviation.

To evaluate workshop interest and barriers to participation, we surveyed the families enrolled in the VIE study (Table 5). Twenty-six families out of 31 answered the appreciation survey: one family could not be reached and 4 others dropped out of the VIE study before the survey was conducted. At the time of the survey, patients’ mean time since diagnosis was 8 months (range: 1 to 14 months) (Table 5). While the diagnoses were not collected, the population admitted in the Division of Hematology-Oncology is highly heterogeneous (Table 1). Because of low participation at the workshops, data from the observation checklist was not collected.

**Table 5:** Demographic characteristics of families who completed the appreciation survey

	Participants n = 26
Relationship with patient, n (%)	
Mother	19 (73%)
Father	6 (23%)
Patient	1 (4%)
Sex of the patient, male n (%)	17 (65%)
Age of patient (years), mean (SD, range)	7.80 (4.99, 1.68–18.09)
Time since diagnosis (months), mean (SD, range)	7.98 (0.81, 1.63–14.23)

SD: Standard deviation.

### Evaluation of the implementation process

This section details the perceptions and opinions obtained from the families and the facilitator. Data were collected using the questionnaires, facilitator activity reports and survey. Results are presented according to each component of the process evaluation. Each qualitative segment could have been included under more than one theme.

#### Procedures for workshop promotion and recruitment

Three themes were identified for the recruitment component: 1) procedure description; 2) impact of recipes and; 3) availability of families. Workshops were promoted using wall posters in strategic locations in the inpatient and outpatient clinics of the Division of Hematology-Oncology. Seventy-three percent (73%) of the families surveyed were aware of the workshops (Table 6). The facilitator visited the families enrolled in the VIE study to promote the workshop of the week. Families attending the common areas were also approached. The total number of families approached during the promotion tours was not documented. The facilitator contacted 16 of the 17 workshop participants during promotion tours. Flyers were also handed to the nurses in the outpatient clinic to encourage promotion of the workshops to families. Nurses and clinical dieticians were also notified each week.

**Table 6:** Preferences in the workshop themes and mode of delivery according to the appreciation survey

	Participants *n* = 26
Interest in recipe tasting	18 (69%)
Interest in content related to foodborne infections	11 (42%)
Awareness of the workshops	19 (73%)
Awareness of the workshops via posters	12 (46%)
Preferred mode of workshop delivery	
Flyers	4 (15%)
Online videos	18 (69%)
Face-to-face workshops	3 (12%)
Videoconference	4 (15%)
No best option	1 (4%)
Most useful theme	
Meal fortification	8 (31%)
Changes in taste during cancer therapy	10 (38%)
Adapting diet to eating-related side effects of treatments	3 (12%)
Mediterranean diet and health	3 (12%)
Planning quick and economic meals	7 (27%)
Nutritional support	3 (12%)
Less useful theme	
Meal fortification	0 (0%)
Changes in taste during cancer therapy	0 (0%)
Adapting diet to eating-related side effects of treatments	8 (31%)
Mediterranean diet and health	5 (19%)
Planning quick and economic meals	6 (23%)
Nutritional support	4 (15%)
None	3 (12%)
Barriers to participation	
Nutrition not a priority	1 (4%)
Theme not related to actual child’s condition	16 (62%)
No other person could stay with the child	15 (58%)
Doctor or health professional could visit during activity	20 (77%)
Scheduled treatment or test during activity	22 (85%)
Unaware of the workshop location	2 (8%)
Too busy	11 (42%)
Other	17 (65%)

#### Reach

Three subthemes emerged from the reach component: 1) impact of low participation; 2) target population and; 3) characteristics of patients. The reach of the population was low as only 1 to 2 participants attended each workshop (Table 3). The facilitator reported that this caused delivery-related difficulties and affected the possibility for participants to interact. Language had a minor impact on the workshop reach: overall, only 4 parents were unable to participate to a workshop because they did not understand or speak French.

Subthemes related to the target population and patients’ characteristics that either facilitated or challenged participation were described using observation notes and survey. Many surveyed parents reported they were cooking at home and that nutrition was a priority for them. Some mothers surveyed had professional cooking (*n* = 3) or a dietician (*n* = 1) education. This was not a barrier for participation but did influence participants’ interests:

(Survey) *One mother reported preferring the treatment-related themes because she had professional cooking training*.

Some parents mentioned that being at the hospital with their spouse would help them attend an activity. Other parents mentioned that the workshops were more relevant for their spouse, suggesting that the reach is not equivalent within a same family:

(Survey) *I am not the one who cooks at home.*

(Survey) *My spouse is more present than me at the hospital*.

Characteristics of patients also influenced parents’ participation and interests. The most stated characteristics were the child’s current health condition related to his treatment, allergies or lactose intolerance, as well as his pickiness. These conditions were mentioned as barriers for recipe tasting or for workshop participation.

#### Dose delivered

Three sub-themes emerged and segments were extracted almost exclusively from the activity report: 1) adaptations of the content and animation; 2) description of the key messages that were not delivered and; 3) reason for not delivering the key messages.

In 7 of the 13 workshops, all the nutritional messages were covered (54%) (Table 4). Messages related to the prevention of foodborne infections were the most omitted. The reasons for not delivering the key messages included the unreceptiveness of participants, oversight from the facilitator, participant prematurely leaving the activity and difficulties performing the recipe.

The adaptation subtheme refers to the strategies used by the facilitator to adapt the messages and animation for the audience. For example, she gave personalized advice to take into account participant’s comments and involved the children in the cooking demonstration. This did not alter the content of the messages delivered. Moreover, during one workshop, the content was adapted in order to address the parent’s questions and knowledge.

#### Contextual factors

Five subthemes have emerged as contextual: 1) location and material; 2) medical or nutritional characteristics of the patient; 3) Logistics and time management; 4) parental presence required elsewhere and; 5) characteristics of the target population.

Difficulties related to the physical location, equipment and noises during the culinary demonstrations were reported as challenges by the facilitator. During the promotional tour, one mother and one nurse reported that it would be more convenient if the workshops would take place on the same floor as the inpatient Division of Hematology-Oncology, rather than on the floor the activity was planned (2 floors below)**:**

(Field notes) *One mother mentioned that she thought this kind of activity was very interesting and that she wanted to participate. However, she could not attend because her child was immunocompromised and she was reluctant to leave her alone. She stated that she would participate if the activity would take place on the same floor as her child’s room.*

Difficulties related to logistics and time management were commonly reported by families. During the promotion tours, many parents stated that they lived far away from the hospital and would not come only to attend a workshop. In the outpatient clinic, parents reported that, when visiting the hospital, they were generally busy with appointments in the morning and wanted to leave as soon as possible. One parent mentioned:

(Field notes) *[Parents] always want to leave before hitting traffic. They corroborated that this is the principal barrier to attend a workshop because they are otherwise interested [in participating to a workshop].*

Lack of time was also a recurrent factor of the target population. Forty-two percent (42%, *n* = 11) of the families surveyed mentioned that being too busy prevented them to participate (Table 6).

Availability and interests of parents were highly affected by patients’ medical condition (Table 6). In the survey, 77% (*n* = 20) of parents stated that expecting a visit from a doctor or a health professional at the time of the workshop would be a barrier for participation. A scheduled test or treatment would also be a barrier for 85% (*n* = 22). For some parents, nutritional difficulties encountered by the child were a motivation to attend, while for others, their management was rather perceived as time-consuming and as a barrier:

(Field note) *A mother stated that she is, in essence, interested, but at the moment, she believes she was being more helpful by focusing on her child’s acute nutritional challenges.*

The need for the parent to be at his child’s bedside was reported in the appreciation survey as a participation barrier by 58% of families (*n* = 15) (Table 6), regardless of the child’s age. The presence of both parents was reported as a facilitator for workshop participation. One mother stated:

(Survey) *We were lucky to be both present [at the hospital] that day: it made it easier to attend the workshop. When only one parent comes [to the hospital] with the child, it is more complicated to attend a workshop [for him/her].*

The contextual factors highlighted the barriers and reasons that complicate parents’ access to the workshops and helped explaining the general low attendance.

#### Fidelity

Four themes have emerged related to fidelity of the implementation process: 1) workshop delivery; 2) recipes; 3) minimization of the burden related to participation and; 4) impact of low participation. The workshops had a mean duration of 51 min (± 13 min, range: 40–90), which was shorter than the planned 60 min (Table 4). The absence of the chef in 69% of the delivered workshops led to a shorter mean duration (46 ± 4 min, range: 40–50).

The facilitator described some difficulties related to the message delivery in the absence of the chef. They principally referred to coordinating the delivery of nutritional messages with the recipe demonstration. Other difficulties reported were a less dynamic or fluid delivery, omission of content and challenges in determining the best moment to answer participants’ questions.

Sixty-nine percent of the surveyed parents (69%, *n* = 18) were interested in tasting the recipes (Table 6). One participant mentioned that the recipe persuaded her to come to the workshop: one mother mentioned the one-pot mac’n’cheese recipe as the main reason explaining her participation. Some parents mentioned the pickiness of their child as a barrier to taste new recipes. However, while the majority of parents stated that tasting was enjoyable, it was not the principal incentive for participation:

(Survey) *[Mother] I would have come even though there was no recipe. However, it was appealing to me.*

It was essential for the research team to minimize the burden associated with participation. On occasions, the facilitator adapted the time and content in order to accommodate participants’ schedule.

#### Dose received

The dose received refers to the exposure and perceived utility of the intervention. In the questionnaires, 71% of participants (12/17) agreed they had acquired knowledge related to every key message (Table 7). Only 13% (2/17) stated they had acquired knowledge for less than half of the key messages. All participants (*n* = 17) would recommend the workshops to other parents. For this component, the subthemes raised from the qualitative data were: 1) delivery mode; 2) interest and receptivity; 3) logistical and organizational context; 4) prior knowledge; 5) utility/non-utility of the workshop related to patient’s condition and; 6) workshop themes.

**Table 7:** Knowledge acquisition and perceived utility of the workshops according to participant questionnaires

	Participants n = 17
Perception of knowledge acquisition	
100% of the key messages	12 (71%)
Equal or more than 50% of the key messages	3 (18%)
Less than 50% of the key message	2 (12%)
Would recommend workshop	100%
Intent to use advices or recipes	100%

When asked about their favorite delivery format, most parents surveyed preferred short web-based video capsules (*n* = 18, 69%, Table 6) in comparison with flyers only, face-to-face or videoconference. Families reported that videos were more appealing than written documentation because they are less time consuming and they can be watched whenever needed.

Data acquired during promotional tours or with the survey showed that parents were interested in nutrition: only one parent stated that healthy eating was not his priority (Table 6). Besides, for 12 of the 13 workshops, the facilitator rated the participants’ level of interest form high to very high (Table 4). In the appreciation survey, many families expressed that workshops could be a nice distraction while being at the hospital:

(Survey) *This kind of activity is very relevant to me because we are often looking for something to do in the hospital.*

In general, there was interest for the activity, but the logistical barriers (e.g. having to stay with the child or living far from the hospital) limited the exposure to the intervention. No pattern in terms of the day (week or week-end) and time (morning or afternoon) of delivery or in the workshop theme was identified in relationship with attendance. Also, families’ preferences were very diverse when asked about what time of the day would be ideal to attend a workshop. However, parents stated that it was easier to attend when the child was hospitalized rather than when he was an outpatient.

The perceived utility of the workshops was influenced by parents’ prior culinary and nutritional knowledge. This was mainly related to foodborne illness prevention as 58% of participants reported not being interested by this specific content (Table 6). These parents stated they had received thorough instructions by the nursing staff and were already applying the principles at home. Conversely, other parents found that reminders of the rules for prevention of foodborne infections were helpful.

The perceived utility was influenced positively or negatively by the child’s medical condition. Some parents reported that their child could eat everything, so they did not perceive the workshops as useful. Other mentioned that their child was picky and that this was a barrier to attend. Parents’ interest towards the activity was also often related to treatment side effects:

(Field notes) *One mother stated that, at the moment, her child was doing well and that she will consider [the workshops] if the child loses weight.*

(Survey) *The utility of the workshops is related to the treatment side effects and the child’s eating habits.*

Thus, 62% of the parents surveyed (*n* = 16/26, Table 6) reported that the workshop theme was not related to the child’s current condition, which was a barrier for participation. The survey showed that “Changes in taste during cancer therapy”, “Meal fortification” and “Planning quick and economic meals” were the most useful workshops for 38, 31 and 27% of the parents, respectively (Table 6). Parents reported that the least useful workshops for them were “Adapting diet to eating-related side effects of treatments” (31%), “Mediterranean diet and health” (23%) and “Planning quick and economic meals” (19%) (Table 6). Among the families who stated that the least useful theme was “Adapting diet to eating-related side effects of treatments”, 25% also reported that their child did not suffer from these side effects. One mother specified that even though she did not find this theme useful, it could be for other parents.

## Discussion

In our study, the process evaluation has identified contextual and reach factors as the main barriers to participation. Indeed, the characteristics of the target population were a subtheme that emerged from these components. Clearly, the contextual factors and the population profile impacted the reach of the intervention. This included greater parents’ availability during the child hospitalization as well as logistics and time constraints.

In Canada, between 2006 and 2010, the number of cancer diagnoses in children under the age of 5 was more than twice of those in older children and adolescents [30]. Young children require parental supervision, a reality that must be considered to assure the success of interventions in pediatric oncology. Also, many children are immunocompromised and cannot leave their hospital room. Side effects, treatment schedules and appointments were also pinpointed as major barriers to participation. Conversely, parents requested access to nutritional information specific to their child’s condition.

It has been reported that, during treatments, parents use a variety of strategies to make the child eat and often force them to eat [31]. Studies showed that from 5 to 60% of children with cancer suffer from malnutrition at one point during their treatments [32, 33]. Here, we found that parents were highly interested in the themes of “Meal fortification” and “Changes in taste during cancer therapy”. Weight loss in a child with cancer is stressful for parents and can disrupt the routine of families [34]. Changes in tastes are also frequent and can modify the child’s eating habits and lead to the consumption of foods high in fat and sodium [5]. This can affect meal ambiance and the child’s quality of life in relation to the symbolic and cultural value of food [35, 36]. Other studies showed that pickiness and cravings were the principal difficulties reported by parents during treatments [1, 2]. In our study, parents highlighted the challenge to find information related to these problems, which explains the high perceived utility of these themes. Depending on participants, the theme “Planning quick and economic meals” was designated as both the most and least useful. It is possible that families who experienced the diagnosis longer ago further valued information related to general healthy eating. However, the small study sample did not allow data stratification to verify this hypothesis.

To our knowledge, this is the first study describing the implementation of an educational nutrition and cooking workshop program taking place early after the diagnosis. An expert consensus of the *Children’s Oncology Group* revealed that the most valuable information for the parents soon after the diagnosis was directly related to the child’s current care including the management of side effects and the prevention of infections [37]. As part of the interdisciplinary VIE study, the psychological intervention *Taking Back Control Together* was developed and refined based on a parents and healthcare professionals’ assessments of the program limitations, benefits and needs for improvement [38]. The qualitative data revealed the need for parents to better manage their distress following the diagnosis in order to be more helpful for their child [38]. This supports findings, including ours, that parents’ interests in knowledge are mainly centered on their child’s medical condition [39].

Raber et al. described a hands-on in-camp culinary intervention destined for children with cancer, survivors and siblings [21]. They also proposed an alternative in-hospital activity. Children in the camp were highly interested in participating to the activity and the reach was high: all the activities reached the maximal participation, in contrast with our results. Differences in the target populations (children vs. parents) and participants’ availability (campers vs. caregivers) can explain the divergent outcomes.

Information collected with the activity reports revealed that 54% of the workshops covered 100% of the nutritional key messages. Low attendance mostly impacted the delivery of the workshops rather than their content. Despite the low attendance, 88% of the 17 participants reported that they had acquired knowledge for more than 50% of the key nutritional messages. While the small number of participants does not allow for efficacy measurement, these numbers offer encouraging data on the educational value of the curriculum.

Basic nutritional knowledge is a prerequisite for behavior changes [40, 41]. Also, adding culinary demonstrations in a nutritional intervention has helped participants to use the given advices and to increase their culinary confidence [42]. Parental involvement in nutrition interventions targeting children is essential because their knowledge and confidence influence the nutritional value of the food offered [43, 44]. Interventions involving only parents were found at least as efficient as the family-based ones for obesity treatment [44].

In the VIE study, the workshops were developed as a complementary resource to clinical follow-up with the concern of not increasing the burden for families. Parents in our study often felt overwhelmed which could explain why their interest in the workshops did not transfer into participation. Based on the results of our feasibility study, we conclude that because of the many barriers encounter by parents, workshops in a face-to-face format are poorly feasible in the context of pediatric oncology. It is possible that using a more accessible mode of delivery would better target this population. However, it has been reported that large amounts of written or verbal information negatively affect retention during treatments for childhood cancer [45].

Accordingly, we propose that the efficacy of web-based initiatives, such as videos, should be tested in this population. For example, a video program was found effective for education in populations of parents with premature infants [46], but has not been tested yet in childhood cancer. Similarly, based on a review of the literature, one of the recommendations issued by Rodgers et al. was ***that “written material, short verbal discussions, and audio recordings of the diagnostic discussion could be used to provide education to pediatric patients newly diagnosed with cancer and to their parents and siblings”[46].*** In the future, assessing the impact of web-based educational programs on knowledge acquisition, food intake and culinary competency would provide a measure of their efficacy.

Our study has some limitations. The majority of participants to the workshops and the VIE study expressed a high level of interest in nutrition, which may not be representative of all families, a well-documented bias of any nutritional intervention. Moreover, given the small sample size, the study representativeness is limited. Also, the facilitator was involved in data collection and analysis, which could potentially introduce bias. Different strategies were used to minimize this caveat including using multiple methods of data collection, gathering the perceptions of participants, non-participants, interveners and of the facilitator, and finally using a strict procedure of qualitative data inter-validity analysis [26]. Furthermore, self-reported barriers could have been subjected to desirability bias although the facilitator specified that the survey aimed to improve access to the activity. In this study, survey administration and discussions with families (field notes) were not recorded and no formal interviews were performed. This could introduce recall bias that was minimized by the facilitator taking notes during the discussion. Also, the heterogeneity of the population surveyed in terms of diagnoses and of time since diagnosis could have influenced the perceived utility or the time available for participation. The use of a validated questionnaire would have increased inter-validity of results. Also, documenting the number of persons approached and patients’ diagnosis for field notes would have allowed a better understanding of the study population.

## Conclusion

This study presents the feasibility of nutrition education and cooking workshops in pediatric oncology, a population that is confronted to complex emotional and organizational challenges. Because the reach was low, we could not conclude on the efficacy of the workshops to increase the perception of knowledge acquisition. However, the process evaluation allowed us to document the need for families to access reliable nutritional information when it is relevant for them. Therefore, there is a need to develop strategies and delivery formats that address the major barriers for participation encountered by this population.

In conclusion, our study showed that nutrition interventions targeting families confronted to childhood cancer should be adapted to improve access to information, reach and delivery. Our results highlighted the importance of process evaluation when developing innovative programs for vulnerable population to ensure they are appropriate for real-life conditions.

## Data Availability

The datasets used and/or analysed during the current study are available from the corresponding author on reasonable request.

## Abbreviations

SD: Standard deviation
VIE: Valorization, Implication, Education
